# Different clinical parameters inform epicardial fat thickness in pre- and post-menopausal women with obstructive sleep apnea

**DOI:** 10.1186/s12905-021-01384-4

**Published:** 2021-06-11

**Authors:** Yong Zhang, Jian Wang, Wen Shui, Zhenxia Zhang, Juan Li, Jin Ma

**Affiliations:** 1 Medical Imaging College, Shanxi Medical University, Taiyuan, 030001 Shanxi China; 2 Department of Ultrasound, First Hospital of Shanxi Medical University, No.85, South Jiefang Road, Yingze District, Taiyuan, 030001 Shanxi China; 3 Department of Respiratory, First Hospital of Shanxi Medical University, Taiyuan, 030001 Shanxi China

**Keywords:** Obstructive sleep apnea, Menopausal, Epicardial fat thickness, Apnea–hypopnea index, Body mass index

## Abstract

**Background:**

Obstructive sleep apnea (OSA) is a sleep-related disorder with breathing difficulties. Previous studies revealed that epicardial fat thickness (EFT) correlates with OSA severity. Interestingly, female patients display a stronger EFT-OSA correlation than males. The purpose of this study is to investigate the relationship between EFT and different clinical characteristics in pre- and post-menopausal women diagnosed with OSA.

**Methods:**

Patients diagnosed with OSA were divided into pre/early peri-menopausal (Group 1) and post/late peri-menopausal (Group 2) according to the menopause status. EFT was obtained from parasternal long-axis echocardiographic images. We also collected general clinical characteristics of patients involved in this study, and performed spearman correlation analysis to explore the correlations between EFT and the general clinical characteristics. We further applied Multiple stepwise linear regression analysis to explore the predictors for EFT in both groups.

**Results:**

A total number of 23 and 59 patients were enrolled in Group 1 and Group 2 respectively. EFT in Group 2 was significantly higher than that of Group 1. In both groups, EFT was positively correlated with apnea–hypopnea index (AHI), percentage of total sleep time when blood oxygen saturation was less than 90% (T90), oxygen desaturation index (ODI) and glucose; while EFT was negatively correlated with mean and lowest SaO_2_ (oxygen saturation) levels. However, EFT was positively correlated with total cholesterol (TC) only in Group 1 and body mass index (BMI) only in Group2, respectively. Multiple stepwise linear regression analysis showed that AHI was independently associated with EFT in Group 1. However, both AHI and BMI were independent predictors of EFT in Group 2.

**Conclusion:**

EFT was notably correlated with menopausal status in women with OSA. AHI was the independent predictor of EFT in women with OSA. BMI was the independent predictor of EFT in post/late peri-menopausal women with OSA.

## Background

Obstructive sleep apnea (OSA) is a sleep-related disorder with breathing difficulties, which is characterized by repetitive collapse of upper respiratory tract during sleep [1]. These obstructive events lead to recurrent intermittent hypoxia and sleep disruption, which can result in cardiovascular and metabolism symptoms. OSA is clinically defined with an occurrence of apnea–hypopnea index (AHI) ≥ 5/h, and the incidence is approximately 22% in men and 17% in women [2]. Menopause status is associated with an increased risk of OSA. It is reported that the prevalence of OSA greatly varies among the pre-versus postmenopausal women from 9 to 30% [3]. Furthermore, a previous study showed that the odds ratios (ODs) for AHI ≥ 5/h in peri-menopausal, peri-menopausal/post-menopausal, and post-menopausal (versus pre-menopausal) individuals were 1.23, 1.80 and 2.60, respectively [4], indicating a tendency of increasing apnea–hypopnea incidence with the progression of menopause. On the other hand, estrogen withdrawal after menopause affects fat distribution, with post-menopausal women having significantly increased visceral fat [5]. Importantly, visceral adipose tissue is an important predictor of metabolic heart disease, which seems to be a greater risk factor than general fat accumulation [6].

Epicardial fat is the largest visceral fat storage situated between the epicardium and pericardium [6]. Epicardial fat thickness (EFT) is defined as the thickness of thoracic adipose tissue external to the parietal pericardium, which can be quantified by echocardiography [6, 7]. There is growing evidence that EFT shows strong correlation with metabolic syndromes, such as blood glucose level, hypertension, diabetes mellitus, and atherosclerosis. Notably, EFT is now considered as a significant cardiovascular risk predictor [6]. Previous studies also revealed that EFT correlates with OSA, and EFT seems to be the most significant predictor of OSA severity [7]. Furthermore, OSA patients with thicker EFT have more chance to develop metabolic syndrome [8]. Interestingly, female OSA patients display a significant stronger correlation between EFT and OSA severity than male patients [9]. Therefore, both EFT and post-menopause status seem to associate with the increased risk of OSA. However, whether there is a relationship between EFT and menopausal status, and which clinical parameters can best predict the EFT in women patients with OSA remain to be further elucidated. In this study, we attempt to investigate the relationship between EFT and different clinical characteristics in pre- and post-menopausal women diagnosed with OSA, with the aim to identify the clinical parameters which can predict the EFT in women patients.

## Methods

### Subject enrollment and ethical approval

**Fig. 1:**
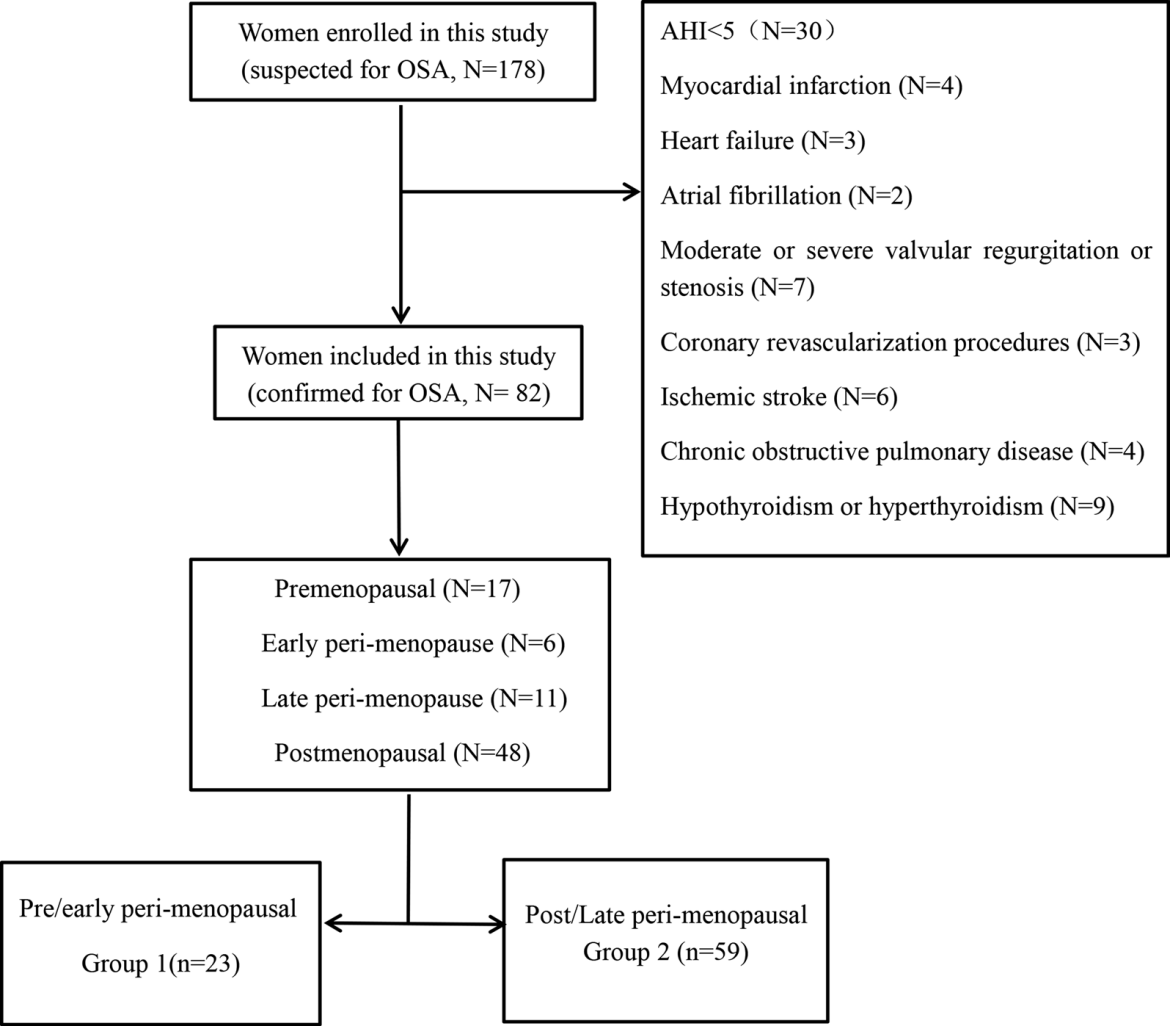
Schematic flowchart of OSA patient enrollment

Women with suspected OSA were consecutively enrolled in the sleep unit in Shanxi Medical University First Hospital from January 2019 to June 2019. All subjects were enrolled after being informed of the details of this study and signing a written consent form. The study was approved by the Institutional Ethics Committee of the Shanxi Medical University First Hospital. No patients enrolled had previous OSA diagnosis. According to OSA diagnosis guideline [10], subjects were divided into OSA (AHI ≥ 5) and non-OSA (AHI < 5) groups. Subject inclusion criteria: (1) Patients without continuous positive airway pressure (CPAP) treatment. Subject exclusion criteria: (1) patients with cardiovascular and cerebrovascular diseases (i.e., myocardial infarction, heart failure, atrial fibrillation, moderate/severe valvular regurgitation or stenosis, coronary revascularization procedures, ischemic stroke, etc.); (2) patients with cardiac dysfunction (left ventricular ejection fraction < 50%); (3) patients with moderate / severe hepatic and renal disease; (4) patients with chronic obstructive pulmonary disease; (5) patients with hypothyroidism/hyperthyroidism; (6) patients with pregnancy; (7) patients with surgical menopause; (8) patients receiving hormone therapy. Figure 1 showed the schematic workflow of the subject enrollment in this study. 182 subjects were initially enrolled, with 82 subjects with OSA diagnosis being included in the final analysis.

### Classification of menopausal status

In this study, we recruited patients diagnosed with OSA, and divided them into pre/early peri-menopausal (Group 1) and post/late peri-menopausal (Group 2). We measured EFT and collected general clinical characteristics in both groups. The menopause status of enrolled subjects was classified by the frequency and regularity of menstrual bleeding as previous described [11]. 4 categories were defined as below: (1) pre-menopause, with no significant change in menstrual cycle (n = 17); (2) early peri-menopausal, with a significant change in menstrual cycle interval and at least one menstrual cycle in the past 3 months (n = 6); (3) late peri-menopausal, with 3 consecutive months of amenorrhea (n = 11); (4) post-menopause, with 12 months of amenorrhea (n = 48) (Fig. 1). Due to the limitation of sample size, pre-menopause and early peri-menopausal patients were defined as Group 1 (n = 23), and late peri-menopausal and post-menopause women were defined as Group 2 (n = 59).

### Clinical and biochemical examination

Waist circumference of an enrolled subject was measured midway between the lower costal margin and iliac crest as described previously in [12]. Weight and height were measured, and Body Mass Index (BMI) was calculated as Weight/Height^2^ [13]. Blood pressure was measured by a mercury sphygmomanometer, after a patient had at least 30 min of rest in a seating position. Blood samples from the peripheral vein were collected during the early follicular phase. Total cholesterol (TC), triglyceride (TG), high-density lipoprotein (HDL), low-density lipoprotein (LDL), and fasting blood glucose were measured by an automatic biochemical analyzer (OLYMPUSAU5400, Japan). Estradiol and follicle-stimulating hormone were measured by an automatic biochemical analyzer (COBAS6000, Germany).

### Echocardiography

**Fig. 2:**
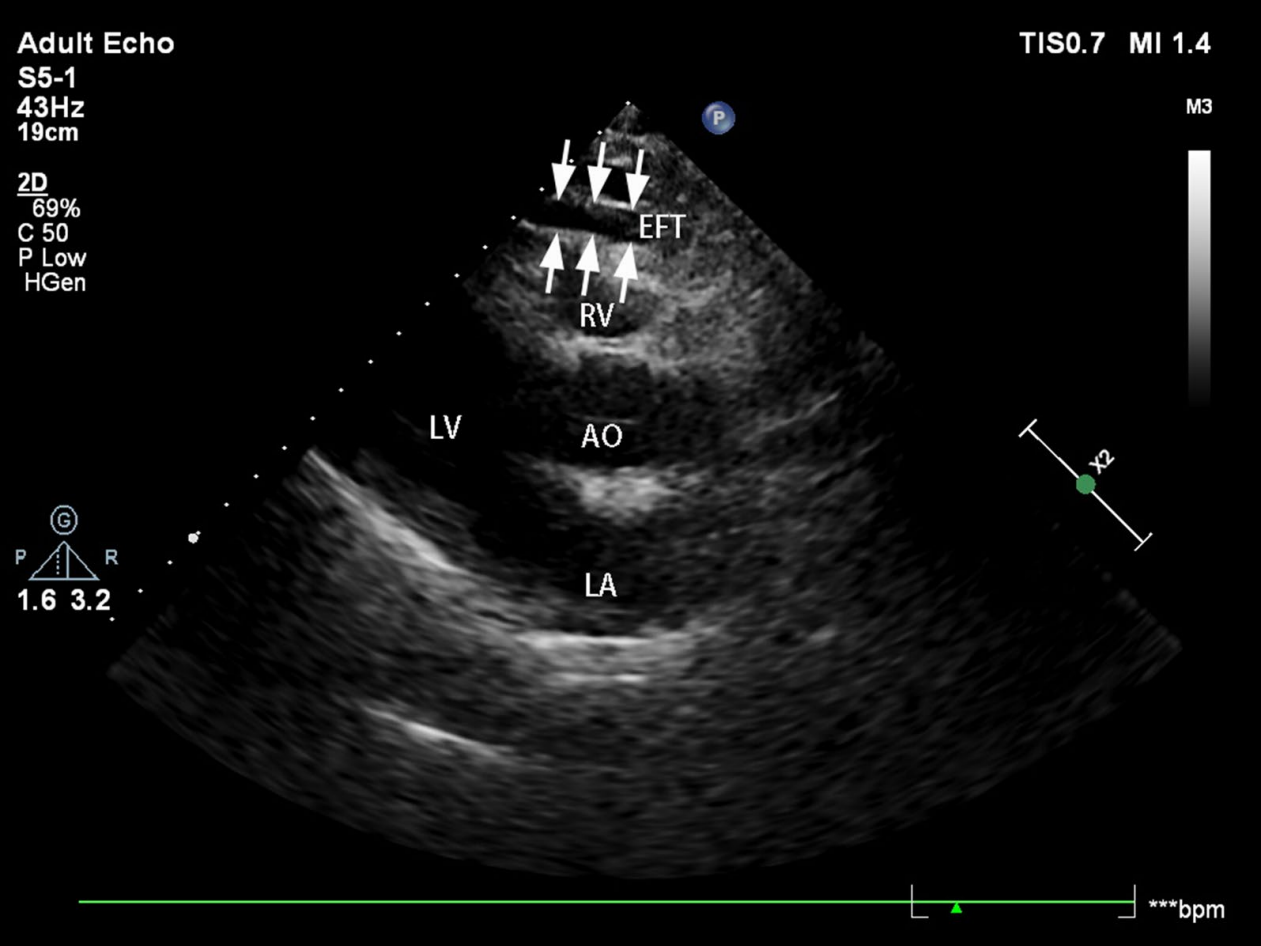
Epicardial fat is an echo-free area in front of the right ventricular free wall, as indicated by the white arrow

All patients underwent transthoracic echocardiographic evaluation according to the recommendations by American Society of Echocardiography [14]. All echocardiograms were recorded by the same experienced operator who was blinded to the identities of subjects in the study. Echocardiography was performed using EPIQ 7C Color Doppler Ultrasound (PHILIPS), with S5-1 probe at the frequency of 1.0–5.0 MHz. EFT was identified as the echo-free space between the outer wall of myocardium and the visceral layer of pericardium. This parameter was measured on the free wall of the right ventricle at end-systole in three cardiac cycles [15]. An example echocardiogram image was shown in Fig. 2.

### Polysomnography

All patients underwent overnight polysomnography (Bond EmblaN-7000; Medcare Flaga, Reykjavik, Iceland) at a sleep laboratory. All the parameters in the sleep recordings including apnea–hypopnea index (AHI), mean oxygen saturation (Mean-SaO_2_), lowest nocturnal oxygen saturation (Lowest-SaO_2_), oxygen desaturation index (ODI), and percentage of total sleep time when blood oxygen saturation was less than 90% (T90), were measured by a skilled technician following American Academic Sleep Medicine (AASM) 2007 criteria. AHI was defined as the number of apnea and hypopnea events per hour of sleep, and a subject with an AHI ≥ 5 was diagnosed as OSA. ODI was defined as the total number of episodes with more than 3% oxyhemoglobin desaturation per hour of sleep. T90 was defined as percentage of total time when  SaO_2_ < 90%.

### Statistical analysis

All data were analyzed using SPSS 22.0 for Windows (IBM, Armonk, NY, USA). Continuous data with normal distribution were presented as means ± standard deviation (SD) and analyzed using the two-tail independent-sample *t*-test. Continuous data with non-normal distribution were presented as medians (Inter-quartile Range, IQR) and analyzed using Mann–Whitney *U*-test. Categorical variables were presented as frequencies (percentages, %) and analyzed using Chi-squared statistic or Fisher’s exact test. Spearman correlation analysis was performed to explore the relationships between EFT and other continuous variables. Multiple stepwise linear regression analysis (forward–backward method) was used to determine EFT predictors. All the parameters such as age, BMI, WC, AHI, mean SaO2, lowest SaO2, T90, ODI, total cholesterol, triglyceride, high-density lipoprotein, low-density lipoprotein, fasting blood glucose, estradiol and follicle-stimulating hormone were included in multiple stepwise linear regression analysis. *P* < 0.05 was considered statistically significant.

## Results

### Clinical characteristics of pre/early peri-menopausal and post/late peri-menopausal OSA patients

A total of 82 OSA patients were included in this study, with a mean age of (51.91 ± 9.00) years. The clinical characteristics were presented in Table 1. Notably, age, BMI, waist circumference, systolic blood pressure, and diastolic blood pressure were all higher in Group 2 compared to Group 1 (*P* < 0.05). Furthermore, the levels of total cholesterol, low-density lipoprotein, glucose, follicle-stimulating hormone, and apnea hypopnea index were significantly increased in Group 2 than Group 1 (*P* < 0.05). In contrast, estradiol level was significantly lower in Group 2 than Group 1 (22.17 [19.32–28.60] vs. 93.17 [72.14–109.0], *P* < 0.001). Furthermore, EFT also showed a significant increase in Group 2 when compared to Group 1 [(3.40 ± 0.93) vs. (2.82 ± 0.83), *P* = 0.011] (Table 1). The above data are consistent with notion that post-menopausal subjects have reduced level of estrogen level, validating our categorization of subjects based on the menopause status. Our data also indicates that post-menopausal OSA patients tend to have a higher level of EFT. However, we did not observe significant difference in the sleeping habits between two groups (Table 2), suggesting that the sleep habits do not change significantly between two menopausal status if the subjects are diagnosed with OSA.

**Table 1:** Characteristics of patients by menopausal status

	Pre/early peri-menopausal (n = 23)	Post/late peri-menopausal (n = 59)	*P* value
*Clinical*			
Age (years)	40.95 ± 8.12	56.19 ± 4.69	< 0.001
BMI (kg/m^2^)	27.0 ± 2.11	28.84 ± 2.91	0.012
Waist circumference (cm)	84.30 ± 5.39	89.31 ± 7.82	0.007
Systolic blood pressure (mm Hg)	129.0 ± 9.0	134.0 ± 10.5	0.046
Diastolic blood pressure (mm Hg)	80.13 ± 7.22	83.12 ± 6.71	0.080
Hypertension frequency (%)	9 (39.1)	29 (49.2)	0.414
Diabetes frequency (%)	2 (8.7)	12 (20.3)	0.329
Smoking frequency (%)	0 (0)	4 (6.8)	0.573
Alcohol frequency (%)	0 (0)	3 (5.1)	0.556
Epicardial fat thickness (mm)	2.82 ± 0.83	3.40 ± 0.93	0.011
*Laboratory*			
Total cholesterol (mmol/L)	4.05 ± 0.65	4.61 ± 0.79	0.003
Triglyceride (mmol/L)	1.59 ± 0.56	1.82 ± 0.89	0.260
Low-density lipoprotein (mmol/L)	2.43 ± 0.51	2.82 ± 0.63	0.01
High-density lipoprotein (mmol/L)	1.17 ± 0.31	1.19 ± 0.25	0.717
Glucose (mmol/L)	4.77 ± 0.11	5.18 ± 0.09	0.009
Estradiol (pmol/L)	90.59 ± 24.02	25.87 ± 10.80	< 0.001
Follicle-stimulating hormone (mIU/mL)	16.29 (5.1–25.7)	86.29 (55.17–100.00)	< 0.001
*Polysomnography*			
Apnea hypopnea index (events/h)	15.3 (9.6–40.1)	26.3 (15.5–50.0)	0.015
Mean SaO_2_ (%)	92.17 ± 5.55	91.63 ± 3.80	0.610
Lowest SaO_2_ (%)	74.17 ± 17.27	71.98 ± 13.38	0.498
T90 (%)	5.93 (17.25)	7.46 (17.72)	0.840
ODI (events/h)	16.7 (8.6–54.0)	25.60 (12.3–47.6)	0.751

Categorical variables are presented in numbers (%), while non-normal and normal distributed continuous data are presented as medians (Inter-quartile range), or mean ± SD, respectively

BMI: Body mass index; Mean SaO_2_: mean oxygen saturation; Lowest SaO_2_: lowest oxygen saturation; T90: percentage of oxygen saturation less than 90%; ODI: oxygen desaturation index

Hypertension diagnosis: Systolic blood pressure (BP) ≥ 140 mmHg or diastolic blood pressure ≥ 90 mmHg or subjects were taking hypertension medication;

Diabetes diagnosis: Diabetes is defined as FPG ≥ 7.0 mmol/L and/or blood glucose ≥ 11.1 mmol/L 2 h after meal or having received diabetes medication

Smoking: Subjects who have never smoked or who have quit smoking for more than 12 months are defined as non-smokers; subjects who have smoked continuously or cumulatively for more than 12 months are defined as smokers

Alcohol: Subjects who have never drunk alcohol or subjects who have abstained from alcohol for ≥ 12 months are defined as non-drinkers; subjects who drink continuously or cumulatively for ≥ 12 months are defined as drinkers

**Table 2:** Sleeping habit comparison between two menopausal status

Habit	Pre/early peri-menopausal (n = 23)	Post/late peri-menopausal (n = 59)	*P* value
Snoring (%)	14 (60.9)	46 (78.0)	0.116
Daytime sleepiness (%)	15 (65.2)	49 (83.1)	0.080
Insomnia (%)	9 (39.1)	13 (22.0)	0.116

### The correlative analysis between biochemical indexes and EFT

We next sought to investigate which biochemical parameters are correlated with EFT in both pre/early peri-menopausal (Group 1) and post/late peri-menopausal (Group 2) patient. We collected general clinical characteristics of patients involved in this study, and performed Spearman correlation analysis. In pre/early peri-menopausal group, positive correlations were observed between EFT and AHI (r = 0.760, *P* < 0.001), T90 (r = 0.562, *P* = 0.005), ODI (r = 0.620, *P* = 0.002), total cholesterol (r = 0.499, *P* = 0.015), and glucose (r = 0.692, *P* < 0.001), whereas EFT was negatively correlated with both mean (r =  − 0.524, *P* = 0.01) and lowest SaO_2_ (r =  − 0.541, *P* = 0.008). However, no significant linear correlation was observed between EFT and age, BMI, waist circumference, triglyceride, LDL, HDL, estradiol, or follicle-stimulating hormone for those patients (Table 3). In post/late peri-menopausal group, there were positive correlations between EFT and BMI (r = 0.312, *P* = 0.016), AHI (r = 0.627, *P* < 0.001), T90 (r = 0.510, *P* < 0.001), ODI (r = 0.599, *P* < 0.001), and glucose (r = 0.312, *P* = 0.016), and a negative correlation was also found for mean SaO_2_ (r =  − 0.345, *P* = 0.007) and lowest SaO_2_ (r =  − 0.489, *P* < 0.001). Similarly, no significant liner correlation was observed between EFT and age, waist circumference, total cholesterol, triglyceride, low-density lipoprotein, high-density lipoprotein, estradiol, or follicle-stimulating hormone (Table 3). Therefore, total cholesterol level is linked with EFT only in pre/early peri-menopausal OSA patient; while BMI seems to contribute to EFT only in post/late peri-menopausal group.

**Table 3:** The linear relationships of epicardial fat thickness and other factors

	Pre/early peri-menopausal (n = 23)			Post/late peri-menopausal (n = 59)		
	*r*	*P*	95% Confidence interval	*r*	*P*	95% Confidence interval
Age	0.092	0.675	(− 0.327,0.483)	0.210	0.111	(− 0.051, 0.456)
BMI	0.323	0.133	(− 0.069,0.647)	**0.312**	**0.016**	**(0.025, 0.581)**
Waist circumference	0.363	0.089	(− 0.097,0.699)	0.200	0.129	(− 0.068, 0.440)
Apnea hypopnea index	**0.760**	** < 0.001**	(0.451,0.904)	**0.627**	**< 0.001**	**(0.429,0.766)**
Mean SaO_2_	**− 0.524**	**0.010**	(− 0.802, − 0.136)	**− 0.345**	**0.007**	**(-0.545,-0.101)**
Lowest SaO_2_	**− 0.541**	**0.008**	**(− 0.819, − 0.110)**	**− 0.489**	**< 0.001**	**(− 0.676, − 0.276)**
T90	**0.562**	**0.005**	**(0.211, 0.764)**	**0.510**	**< 0.001**	**(0.278, 0.697)**
ODI	**0.620**	**0.002**	**(0.200, 0.830)**	**0.599**	**< 0.001**	**(0.392, 0.756)**
Total cholesterol	**0.499**	**0.015**	(0.123, 0.793)	0.130	0.325	(− 0.141,0.367)
Triglyceride	0.211	0.335	(− 0.250,0.628)	0.214	0.103	(− 0.075, 0.495)
Low-density lipoprotein	0.113	0.609	(− 0.352,0.534)	0.153	0.248	(− 0.133, 0.411)
High-density lipoprotein	− 0.149	0.499	(− 0.523,0.237)	− 0.081	0.543	(− 0.344, 0.205)
Glucose	**0.692**	**< 0.001**	**(0.358, 0.882)**	**0.312**	**0.016**	**(0.075, 0.507)**
Estradiol	− 0.050	0.821	(− 0.462,0.415)	− 0.126	0.340	(− 0.359, 0.122)
Follicle-stimulating hormone	0.187	0.393	(− 0.236,0.602)	0.164	0.216	(-0.122,0.421)

Bolded numbers indicate linear relationships of statistical significance (*P* < 0.05)

BMI: Body mass index; Mean SaO_2_: mean oxygen saturation; Lowest SaO_2_: lowest oxygen saturation; T90: percentage of oxygen saturation less than 90%; ODI: oxygen desaturation index

### Multiple stepwise liner regression analysis reveals the predictors of EFT

Next, we performed multiple stepwise liner regression analysis to identify the predictors of EFT in both groups. Our analysis showed that AHI and BMI were independent predictors of EFT for post/late peri-menopausal women. For pre/early peri-menopausal women, AHI was the only independent predictor of EFT (Table 4).

**Table 4:** Stepwise multiple linear regression analysis of factors associated with epicardial fat thickness

	Beta	*t*	*P*	Beta 95%CI
*Pre/early peri-menopausal*				
Apnea hypopnea index	0.876	8.322	**< 0.001**	(0.026, 0.043)
*Post/late peri-menopausal*				
Apnea hypopnea index	0.691	8.029	**< 0.001**	(0.020, 0.034)
BMI	0.365	4.241	**< 0.001**	(0.062, 0.173)

BMI: Body mass index

## Discussion

In this study, we explored the relationship between EFT and different clinical characteristics in pre- and post-menopausal women with OSA diagnosis. To our knowledge, this is the first research to probe the relationship of different parameters contributing to the EFT in OSA-diagnosed women according to menopause status. In this group of patients, our correlation analysis demonstrated that regardless of the menopause status, EFT was positively correlated with AHI, T90, ODI and glucose level. Our data also indicate that AHI was the universal predictor of EFT in both pre/early peri-menopausal and post/late peri-menopausal women with OSA. However, BMI was the independent predictor of EFT only for post/late peri-menopausal women with OSA. Therefore, our study suggests that total cholesterol level seems to contribute to the EFT only in pre/early peri-menopausal OSA patient; while BMI is a potential manipulative parameter to reduce EFT and the incidence of OSA in post-menopausal women.

EFT is considered as a cardiometabolic risk factor of OSA, which is highly correlated with OSA severity and was a statistically-significant independent predictor of AHI in obesity-related OSA patients [7]. ODI is an independent predictor of thicker epicardial fat in non-obese OSA patients when compared to healthy controls [16]. Moreover, body fat deposition and epicardial fat seem to show strong association with metabolic syndrome development in OSA patients [8]. In a previous study, Akilli et al. [9] were the first to determine the effect of gender differences on EFT, where they found that epicardial adipose tissue was thicker in severe OSA women than those with normal and mild OSA. However, such an OSA severity-EFT association was not observed in males. suggesting that EFT may be a more valuable parameter in the evaluation of OSA severity in women than in men.

It is worth noting that no previous studies have investigated the correlation between menopausal status and EFT in OSA women. In our study, we found that EFT was significantly higher in post/late peri-menopausal OSA patients in comparison to pre/early peri-menopausal patients. Our findings were consistent with a recent study by Khoudary et al. [17, 18] in which post/late peri-menopausal obese women displayed greater amounts of epicardial, paracardial and total heart adipose tissue than premenopausal and early peri-menopausal obese women, which was independent of age, obesity, race, smoking, and other factors, indicating menopause status can affect the epicardial fat accumulation. Moreover, Fernández et al. [19] showed that EFT was thicker in postmenopausal women with metabolic syndrome compared to those without metabolic syndrome. All these data supported the notion that there is a strong association between menopausal status and EFT.

In the present study, we further analyzed the correlations between EFT and different clinical parameters in pre- and post-menopausal OSA patients. We showed that AHI was a common independent predictor of EFT in both groups, whereas BMI was an independent predictor of EFT in post/late peri-menopausal women. A previous study demonstrated that AHI is considered as a potential marker for postmenopausal OSA women without hormonal replacement therapy [20]. Notably, high BMI is considered as a main independent risk factor for postmenopausal women [21]. Additionally, Li et al. [22] reported a significant BMI-snoring association, and a lower BMI could be a protective factor for premenopausal women in OSA. Altogether, our data and these studies altogether suggests a close relevance of BMI in postmenopausal OSA women and that BMI can be manipulative parameter to ameliorate OSA. On the other hand, Cetin et al. [23] showed that continuous positive airway pressure therapy which uses a machine to help a person with OSA breathe more easily is able to reduce EFT. Therefore, EFT and the breathing quality during the sleep may influence each other in a feedback loop. Some other studies suggested that hormone therapy can be used for postmenopausal women in alleviating epicardial fat and OSA [24]. However, further comprehensive studies are needed to fully evaluate the efficacy of hormone therapy on epicardial fat and OSA development.

We also recognize several limitations in our study. First, the sample size was small and the study was cross-sectional so that a cause-effect conclusion was not able to be drawn. Second, OSA patients with thicker EFT have more chance to develop metabolic syndrome [8], it is worth further surveying the correlation of metabolic syndrome with different menopausal status in OSA. Third, it will be informative to investigate the impact of different OSA treatments (such as PAP therapy) on EFT in relationship with the menopausal status. Our study provides a basis for future longitudinal, multicenter study with large sample size to further explore and validate the contribution of different clinical parameters to EFT and OSA in women with different menopausal status.

## Conclusions

Our study shows that EFT was positively correlated with post-menopausal OSA and can be as a risk factor for assessing OSA. Particularly, our finding suggest that BMI is a potential predictor of EFT in post-menopausal women with OSA. A large sample size will be necessary to further validate the contribution of different clinical parameters to EFT and OSA in women with different menopausal status.

## Data Availability

The datasets during and/or analyzed during the current study is available from the corresponding author on reasonable request.

## Abbreviations

OSA: Obstructive sleep apnea
EFT: Epicardial fat thickness
AHI: Apnea–hypopnea index
ODI: Oxygen desaturation index
SaO_2_: Oxygen saturation
CPAP: Continuous positive airway pressure
BMI: Body mass index
T90: Percentage of total sleep time when blood oxygen saturation was less than 90%
